# *N*-(4-Fluorobenzoyl)-2-hydroxy-4-methyl­benzohydrazide

**DOI:** 10.1107/S1600536808029292

**Published:** 2008-09-17

**Authors:** Hai-Mei Feng, Xin Wang, Ke-Wei Lei

**Affiliations:** a State Key Laboratory Base of Novel Functional Materials and Preparation Science, Institute of Solid Materials Chemistry, Faculty of Materials Science and Chemical Engineering, Ningbo University, Ningbo 315211, People’s Republic of China; b Zhejiang Textile and Fashion College, Ningbo 315211, People’s Republic of China

## Abstract

In the title compound, C_15_H_13_FN_2_O_3_, the aromatic rings are aligned at an angle of 10.15 (3)°. The mol­ecules are packed with π–π stacking inter­actions [mean inter­planar distances of 3.339 (2) and 3.357 (3) Å] and the crystal structure is stabilized by inter­molecular N—H⋯O and O—H⋯O hydrogen bonds.  An intramolecular N—H⋯O interaction also occurs.

## Related literature

For background on the chemistry of salicylic acid, see: Dou *et al.* (2006 ▶). For related compounds, see: John *et al.* (2005 ▶, 2006 ▶); Liu *et al.* (2001 ▶); Majumder *et al.* (2006 ▶); Moon *et al.* (2006 ▶).
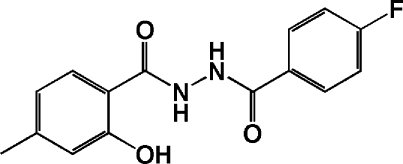


## Experimental

### 

#### Crystal data

C_15_H_13_FN_2_O_3_
*M* *_r_* = 288.27
Triclinic, 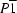

*a* = 7.0969 (13) Å
*b* = 7.2994 (14) Å
*c* = 13.701 (3) Å
α = 102.854 (2)°
β = 97.754 (3)°
γ = 105.538 (1)°
*V* = 652.2 (2) Å^3^
*Z* = 2
Mo *K*α radiation
μ = 0.11 mm^−1^
*T* = 296 (2) K
0.54 × 0.30 × 0.25 mm

#### Data collection

Bruker APEXII diffractometer
Absorption correction: none
4591 measured reflections
2274 independent reflections
2090 reflections with *I* > 2σ(*I*)
*R*_int_ = 0.024

#### Refinement

*R*[*F* ^2^ > 2σ(*F* ^2^)] = 0.047
*wR*(*F* ^2^) = 0.139
*S* = 1.02
2274 reflections
191 parameters
H-atom parameters constrained
Δρ_max_ = 0.39 e Å^−3^
Δρ_min_ = −0.45 e Å^−3^

### 

Data collection: *APEX2* (Bruker, 2007 ▶); cell refinement: *SAINT* (Bruker, 2007 ▶); data reduction: *SAINT*; program(s) used to solve structure: *SHELXS97* (Sheldrick, 2008 ▶); program(s) used to refine structure: *SHELXL97* (Sheldrick, 2008 ▶); molecular graphics: *SHELXTL* (Sheldrick, 2008 ▶); software used to prepare material for publication: *SHELXTL*.

## Supplementary Material

Crystal structure: contains datablocks global, I. DOI: 10.1107/S1600536808029292/ng2492sup1.cif

Structure factors: contains datablocks I. DOI: 10.1107/S1600536808029292/ng2492Isup2.hkl

Additional supplementary materials:  crystallographic information; 3D view; checkCIF report

## Figures and Tables

**Table 1:** Selected bond angles (°)

O1—C6—C5	120.01 (13)
O1—C6—C7	119.48 (13)
O2—C8—N1	121.54 (13)
O2—C8—C7	122.52 (13)
N1—C8—C7	115.94 (12)
O3—C9—N2	120.31 (14)
O3—C9—C10	122.26 (13)
N2—C9—C10	117.42 (12)

**Table 2:** Hydrogen-bond geometry (Å, °)

*D*—H⋯*A*	*D*—H	H⋯*A*	*D*⋯*A*	*D*—H⋯*A*
N1—H1*D*⋯O1	0.86	1.92	2.6224 (19)	139
O1—H1*E*⋯O3^i^	0.82	1.88	2.7035 (18)	177
N2—H2*A*⋯O2^ii^	0.86	2.11	2.9079 (19)	154

Symmetry codes: (i) 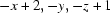; (ii) 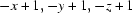.

**Fig. 1.**
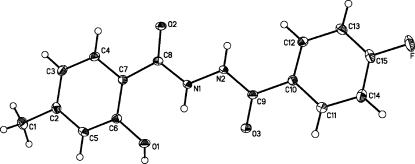
The structure of (I), showing 30% probability displacement ellipsoids and the atom-numbering scheme.

**Fig. 2.**
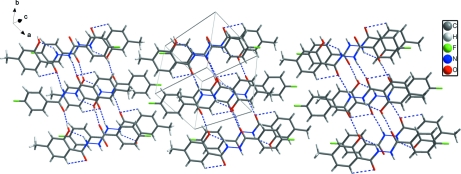
A view of π–π stacking of (I) and H bonds.

## supplementary crystallographic information

### Comment

The chemistry of salicylic acid has attracted the interest of reseachers since 1860s in the application area of skin science. After a long period, investigations in this area have received a new impulse (Dou *et al.*, 2006) and recently there has been notable progress especially regarding the synthesis of new derivatives. *N*-acylsalicylhydrazide is one of the important kind, which have been used extensively as ligands in the field of coordination chemistry. Some of the reasons are that the intramolecular hydrogen bond between the O and N atoms plays an important role in the formation of metal complexes, and the *N*-acylsalicylhydrazide compounds show photoluminescence in the solid state by proton transfer from the O atom to the N atom (Majumder *et al.*, 2006). There several this kind of ligand have been reported, such as *N*-phenylsalicylhydrazidate (Liu *et al.*, 2001), *N*-(2-methylpropanoyl)salicylhydrazide (John *et al.*, 2005), *N*-cyclohexanoylsalicylhydrazidate (John *et al.*, 2006), *N*-3-phenyl-*trans*-2-propenoylsalicylhydrazide (Moon *et al.*, 2006) and so on. With the aim of gaining a deeper insight into the structural aspects responsible for the fluorescent properties in the solid state and crystallographic analysis of the title compound (I), has been carried out and the results are presented in this paper.

The molecular structure of (I), C_15_H_13_FN_2_O_3_, is illustrated in Fig. 1. The bond length and bond angle in (I) are within normal ranges. The bond distances between C—O of carbonyl are significantly shorter than C6—O1 bond distances (Table 1). Atom O1, O2, N1 and N2 are nearly coplanar with the plane of benzene rings that contain C2–C7. The O3 atomic deviation is 0.394 (2) Å from the plane of benzene rings that contain C10–C15 and 0.703 (2) Å from the plane of benzene rings that contain C2–C7. The dihedral angel between the two planes of benzene rings is 10.15 (3)°.

The mean interplanar distance of 3.339 (2) Å between the plane of benzene rings that contain C2–C7 and 3.357 (3) Å between the plane of benzene rings that contain C10–C15 respectively suggests that the ligands are engaged in π–π stacking interactions with a offset face-to-face style. The molecular conformation is characterized by an N—H···O and C—H···O hydrogen bonds and the crystal packing is stabilized by N—H···O and O—H···O hydrogen bonds(Fig. 2).

### Experimental

4-fluorobenzoyl chloride (0.795 g, 5 mmol) and 2-hydroxy-4-methylbenzohydrazide (0.830 g, 5 mmol) were added to 30 ml of DMF solution with an external ice–water bath. When 0.607 g (6 mmol) of triethylamine was added, a white suspension immediately appeared. The suspension was then filtered. The left solution was volume reduced to about one-third on rotary evaporator. After 7 days crystals of the title compound were obtained from the left solution. Yield: 92.2%. Melting point: 217–226 °C. Calcd. for C_15_H_13_FN_2_O_3_: C, 62.50; H, 4.51; N, 9.72; Found: C, 62.24; H, 4.55; N, 9.65%

### Refinement

All H atoms were placed in geometrically idealized positions and constrained to ride on their parent atoms (C—H = 0.93 Å; N—H = 0.86 Å; O—H = 0.82 Å) and *U*_iso_(H) values weren taken to be equal to 1.2 *U*_eq_(C, N) and 1.5Ueq(O).

### Crystal data

**Table:**

C_15_H_13_FN_2_O_3_	*Z* = 2
*M**_r_* = 288.27	*F*(000) = 300
Triclinic, *P*1	*D*_x_ = 1.468 Mg m^−^^3^
Hall symbol: -P 1	Melting point = 490–499 K
*a* = 7.0969 (13) Å	Mo *K*α radiation, λ = 0.71073 Å
*b* = 7.2994 (14) Å	Cell parameters from 6530 reflections
*c* = 13.701 (3) Å	θ = 1.6–27.6°
α = 102.854 (2)°	µ = 0.11 mm^−^^1^
β = 97.754 (3)°	*T* = 296 K
γ = 105.538 (1)°	Block, colourless
*V* = 652.2 (2) Å^3^	0.54 × 0.30 × 0.25 mm

### Data collection

**Table:**

Bruker Kappa APEXII diffractometer	2090 reflections with *I* > 2σ(*I*)
Radiation source: fine-focus sealed tube	*R*_int_ = 0.024
graphite	θ_max_ = 25.0°, θ_min_ = 1.6°
Detector resolution: 0 pixels mm^-1^	*h* = −8→8
ω scans	*k* = −8→8
4591 measured reflections	*l* = −16→16
2274 independent reflections	

### Refinement

**Table:**

Refinement on *F*^2^	Primary atom site location: structure-invariant direct methods
Least-squares matrix: full	Secondary atom site location: difference Fourier map
*R*[*F*^2^ > 2σ(*F*^2^)] = 0.047	Hydrogen site location: inferred from neighbouring sites
*wR*(*F*^2^) = 0.139	H-atom parameters constrained
*S* = 1.02	*w* = 1/[σ^2^(*F*_o_^2^) + (0.093*P*)^2^ + 0.2642*P*] where *P* = (*F*_o_^2^ + 2*F*_c_^2^)/3
2274 reflections	(Δ/σ)_max_ = 0.001
191 parameters	Δρ_max_ = 0.39 e Å^−^^3^
0 restraints	Δρ_min_ = −0.45 e Å^−^^3^

### Special details

**Table:**

Geometry. All e.s.d.'s (except the e.s.d. in the dihedral angle between two l.s. planes) are estimated using the full covariance matrix. The cell e.s.d.'s are taken into account individually in the estimation of e.s.d.'s in distances, angles and torsion angles; correlations between e.s.d.'s in cell parameters are only used when they are defined by crystal symmetry. An approximate (isotropic) treatment of cell e.s.d.'s is used for estimating e.s.d.'s involving l.s. planes.
Refinement. Refinement of *F*^2^ against ALL reflections. The weighted *R*-factor *wR* and goodness of fit *S* are based on *F*^2^, conventional *R*-factors *R* are based on *F*, with *F* set to zero for negative *F*^2^. The threshold expression of *F*^2^ > σ(*F*^2^) is used only for calculating *R*-factors(gt) *etc*. and is not relevant to the choice of reflections for refinement. *R*-factors based on *F*^2^ are statistically about twice as large as those based on *F*, and *R*- factors based on ALL data will be even larger.

### Fractional atomic coordinates and isotropic or equivalent isotropic displacement parameters (Å^2^)

**Table:**

	*x*	*y*	*z*	*U*_iso_*/*U*_eq_	
C1	1.5146 (3)	0.7327 (3)	0.90895 (14)	0.0382 (4)	
H1A	1.5688	0.6263	0.9116	0.057*	
H1B	1.4846	0.7824	0.9741	0.057*	
H1C	1.6105	0.8366	0.8931	0.057*	
C2	1.3255 (2)	0.6585 (2)	0.82719 (12)	0.0275 (4)	
C3	1.1619 (2)	0.7274 (2)	0.83934 (12)	0.0291 (4)	
H3A	1.1679	0.8211	0.8990	0.035*	
C4	0.9911 (2)	0.6566 (2)	0.76276 (12)	0.0256 (4)	
H4A	0.8839	0.7043	0.7722	0.031*	
C5	1.3107 (2)	0.5166 (2)	0.73751 (12)	0.0260 (4)	
H5A	1.4184	0.4696	0.7285	0.031*	
C6	1.1384 (2)	0.4435 (2)	0.66103 (11)	0.0227 (3)	
C7	0.9748 (2)	0.5153 (2)	0.67156 (11)	0.0215 (3)	
C8	0.7818 (2)	0.4502 (2)	0.59554 (11)	0.0210 (3)	
C9	0.5611 (2)	0.0444 (2)	0.37524 (11)	0.0225 (3)	
C10	0.3744 (2)	−0.0450 (2)	0.29516 (12)	0.0228 (4)	
C11	0.3805 (2)	−0.1729 (2)	0.20387 (12)	0.0292 (4)	
H11A	0.4995	−0.1983	0.1948	0.035*	
C12	0.1944 (2)	−0.0106 (2)	0.30968 (12)	0.0258 (4)	
H12A	0.1892	0.0725	0.3708	0.031*	
C13	0.0234 (2)	−0.1002 (2)	0.23314 (13)	0.0298 (4)	
H13A	−0.0972	−0.0786	0.2422	0.036*	
C14	0.2107 (3)	−0.2622 (3)	0.12657 (13)	0.0332 (4)	
H14A	0.2140	−0.3465	0.0654	0.040*	
C15	0.0363 (2)	−0.2216 (2)	0.14356 (13)	0.0311 (4)	
F	−0.13083 (16)	−0.30700 (16)	0.06798 (8)	0.0459 (3)	
N1	0.76066 (18)	0.30055 (19)	0.51310 (10)	0.0239 (3)	
H1D	0.8590	0.2550	0.5049	0.029*	
N2	0.58263 (18)	0.21862 (18)	0.44102 (9)	0.0223 (3)	
H2A	0.4916	0.2760	0.4386	0.027*	
O1	1.12712 (16)	0.30064 (17)	0.57426 (8)	0.0287 (3)	
H1E	1.1820	0.2223	0.5895	0.043*	
O2	0.64816 (15)	0.52694 (16)	0.60736 (8)	0.0270 (3)	
O3	0.69450 (16)	−0.03614 (17)	0.38199 (9)	0.0320 (3)	

### Atomic displacement parameters (Å^2^)

**Table:**

	*U*^11^	*U*^22^	*U*^33^	*U*^12^	*U*^13^	*U*^23^
C1	0.0302 (9)	0.0413 (10)	0.0360 (9)	0.0091 (8)	−0.0048 (7)	0.0057 (8)
C2	0.0260 (8)	0.0262 (8)	0.0273 (8)	0.0051 (6)	−0.0005 (6)	0.0084 (6)
C3	0.0343 (9)	0.0250 (8)	0.0252 (8)	0.0118 (7)	0.0003 (7)	0.0014 (6)
C4	0.0285 (8)	0.0245 (8)	0.0259 (8)	0.0134 (6)	0.0045 (6)	0.0056 (6)
C5	0.0204 (8)	0.0303 (8)	0.0308 (8)	0.0111 (6)	0.0054 (6)	0.0111 (7)
C6	0.0243 (7)	0.0233 (7)	0.0228 (7)	0.0099 (6)	0.0049 (6)	0.0074 (6)
C7	0.0222 (8)	0.0202 (7)	0.0231 (8)	0.0085 (6)	0.0026 (6)	0.0071 (6)
C8	0.0217 (7)	0.0220 (7)	0.0225 (7)	0.0102 (6)	0.0052 (6)	0.0079 (6)
C9	0.0229 (8)	0.0233 (8)	0.0244 (7)	0.0119 (6)	0.0056 (6)	0.0064 (6)
C10	0.0231 (8)	0.0194 (7)	0.0251 (8)	0.0068 (6)	0.0023 (6)	0.0057 (6)
C11	0.0261 (8)	0.0307 (9)	0.0293 (8)	0.0102 (7)	0.0042 (6)	0.0043 (7)
C12	0.0263 (8)	0.0201 (7)	0.0310 (8)	0.0089 (6)	0.0043 (6)	0.0056 (6)
C13	0.0226 (8)	0.0249 (8)	0.0417 (9)	0.0074 (6)	0.0017 (7)	0.0114 (7)
C14	0.0371 (9)	0.0316 (9)	0.0244 (8)	0.0078 (7)	0.0009 (7)	0.0015 (7)
C15	0.0273 (8)	0.0267 (8)	0.0333 (9)	0.0027 (6)	−0.0064 (7)	0.0107 (7)
F	0.0361 (6)	0.0447 (7)	0.0410 (6)	0.0040 (5)	−0.0165 (5)	0.0046 (5)
N1	0.0192 (6)	0.0269 (7)	0.0242 (7)	0.0128 (5)	−0.0015 (5)	0.0011 (5)
N2	0.0184 (6)	0.0238 (7)	0.0241 (6)	0.0112 (5)	−0.0011 (5)	0.0024 (5)
O1	0.0284 (6)	0.0353 (6)	0.0252 (6)	0.0208 (5)	0.0023 (4)	0.0026 (5)
O2	0.0255 (6)	0.0305 (6)	0.0271 (6)	0.0171 (5)	0.0025 (4)	0.0032 (5)
O3	0.0293 (6)	0.0300 (6)	0.0353 (6)	0.0180 (5)	−0.0014 (5)	0.0004 (5)

### Geometric parameters (Å, °)

**Table:**

C1—C2	1.511 (2)	C9—N2	1.342 (2)
C1—H1A	0.9601	C9—C10	1.487 (2)
C1—H1B	0.9601	C10—C12	1.397 (2)
C1—H1C	0.9601	C10—C11	1.398 (2)
C2—C5	1.390 (2)	C11—C14	1.388 (2)
C2—C3	1.399 (2)	C11—H11A	0.9300
C3—C4	1.385 (2)	C12—C13	1.388 (2)
C3—H3A	0.9300	C12—H12A	0.9300
C4—C7	1.401 (2)	C13—C15	1.375 (3)
C4—H4A	0.9300	C13—H13A	0.9300
C5—C6	1.391 (2)	C14—C15	1.385 (3)
C5—H5A	0.9300	C14—H14A	0.9300
C6—O1	1.3732 (19)	C15—F	1.3614 (18)
C6—C7	1.407 (2)	N1—N2	1.3875 (17)
C7—C8	1.494 (2)	N1—H1D	0.8600
C8—O2	1.2351 (18)	N2—H2A	0.8600
C8—N1	1.345 (2)	O1—H1E	0.8200
C9—O3	1.2451 (19)		
			
C2—C1—H1A	109.5	O3—C9—C10	122.26 (13)
C2—C1—H1B	109.5	N2—C9—C10	117.42 (12)
H1A—C1—H1B	109.5	C12—C10—C11	119.65 (14)
C2—C1—H1C	109.5	C12—C10—C9	122.36 (14)
H1A—C1—H1C	109.5	C11—C10—C9	117.95 (13)
H1B—C1—H1C	109.5	C14—C11—C10	120.64 (15)
C5—C2—C3	118.40 (14)	C14—C11—H11A	119.7
C5—C2—C1	119.88 (15)	C10—C11—H11A	119.7
C3—C2—C1	121.71 (15)	C13—C12—C10	120.17 (15)
C4—C3—C2	120.21 (14)	C13—C12—H12A	119.9
C4—C3—H3A	119.9	C10—C12—H12A	119.9
C2—C3—H3A	119.9	C15—C13—C12	118.47 (15)
C3—C4—C7	122.03 (14)	C15—C13—H13A	120.8
C3—C4—H4A	119.0	C12—C13—H13A	120.8
C7—C4—H4A	119.0	C15—C14—C11	117.78 (15)
C2—C5—C6	121.48 (14)	C15—C14—H14A	121.1
C2—C5—H5A	119.3	C11—C14—H14A	121.1
C6—C5—H5A	119.3	F—C15—C13	118.40 (15)
O1—C6—C5	120.01 (13)	F—C15—C14	118.34 (15)
O1—C6—C7	119.48 (13)	C13—C15—C14	123.26 (15)
C5—C6—C7	120.50 (14)	C8—N1—N2	121.02 (12)
C4—C7—C6	117.33 (14)	C8—N1—H1D	119.5
C4—C7—C8	116.67 (13)	N2—N1—H1D	119.5
C6—C7—C8	125.97 (14)	C9—N2—N1	115.98 (12)
O2—C8—N1	121.54 (13)	C9—N2—H2A	122.0
O2—C8—C7	122.52 (13)	N1—N2—H2A	122.0
N1—C8—C7	115.94 (12)	C6—O1—H1E	109.5
O3—C9—N2	120.31 (14)		

### Hydrogen-bond geometry (Å, °)

**Table:**

*D*—H···*A*	*D*—H	H···*A*	*D*···*A*	*D*—H···*A*
N1—H1D···O1	0.86	1.92	2.6224 (19)	139.
O1—H1E···O3^i^	0.82	1.88	2.7035 (18)	177.
N2—H2A···O2^ii^	0.86	2.11	2.9079 (19)	154.
C4—H4A···O2	0.93	2.47	2.797 (2)	101.

Symmetry codes:  (i) −*x*+2, −*y*, −*z*+1; (ii) −*x*+1, −*y*+1, −*z*+1.
